# Novel IncR/IncP6 Hybrid Plasmid pCRE3-KPC Recovered from a Clinical KPC-2-Producing Citrobacter braakii Isolate

**DOI:** 10.1128/mSphere.00891-19

**Published:** 2020-03-25

**Authors:** Dandan Dong, Ziqiang Mi, Dujun Li, Mingming Gao, Nan Jia, Manli Li, Yigang Tong, Xianglilan Zhang, Yuanqi Zhu

**Affiliations:** a Department of Clinical Laboratory, The Affiliated Hospital of Qingdao University, Qingdao, China; b State Key Laboratory of Pathogen & Biosecurity, Beijing Institute of Microbiology & Epidemiology, Beijing, China; c Department of Laboratory Medicine, Yeda Hospital of Yantai City, Yantai, China; d College of Information Science and Technology, Beijing University of Chemical Technology, Beijing, China; JMI Laboratories

**Keywords:** *Citrobacter braakii*, *bla*_KPC-2_, IncR, IncP6, plasmid, transposon

## Abstract

Klebsiella pneumoniae carbapenemase (KPC)-producing *Enterobacteriaceae* have become widespread in hospitals and the environment. Here, we describe a *bla*_KPC-2_-carrying plasmid called pCRE3-KPC, which was recovered from a clinical multidrug-resistant Citrobacter braakii CRE3 strain in China. The complete nucleotide sequence of pCRE3-KPC was determined by combining MiSeq and MinION sequencing and then compared with those of three related plasmids. Plasmid conjugal transfer and electroporation tests, modified carbapenem inactivation method, and bacterial antimicrobial susceptibility test were carried out. We compared this plasmid with three related plasmids to verify that the backbone of pCRE3-KPC was composed of the backbones of the IncR plasmid and IncP6 plasmid. Further bioinformatics analysis showed that pCRE3-KPC carried two resistance-related regions (the *bla*_KPC-2_ gene cluster and the *aacC2*-*tmrB*-related region). The *aacC2*-*tmrB*-related region included two novel insertion sequences (IS*Cfr28* and IS*Cfr16*).

**IMPORTANCE** Reports of human-pathogenic C. braakii strains, especially of strains showing resistance to carbapenems, are rare. To the best of our knowledge, our results represent the first detection of carbapenemase gene *bla*_KPC-2_ in C. braakii strains. In addition, we have studied detailed genetic characteristics of the novel IncR/IncP6 hybrid plasmid pCRE3-KPC, which was isolated from a clinical multidrug-resistant Citrobacter braakii CRE3 strain. Our results may provide further insight into the horizontal transfer of multidrug resistance genes in bacteria and into the genomic diversity and molecular evolution of plasmids.

## Abstract (precis)

Reports of human-pathogenic C. braakii strains, especially of strains showing resistance to carbapenems, are rare. To the best of our knowledge, our results represent the first detection of carbapenemase gene *bla*_KPC-2_ in C. braakii strains. In addition, we have studied detailed genetic characteristics of the novel IncR/IncP6 hybrid plasmid pCRE3-KPC, which was isolated from a clinical multidrug-resistant Citrobacter braakii CRE3 strain. Our results may provide further insight into the horizontal transfer of multidrug resistance genes in bacteria and into the genomic diversity and molecular evolution of plasmids.

## INTRODUCTION

Klebsiella pneumoniae strains that produce K. pneumoniae carbapenemase (KPC) were initially identified in the United States in 2001 (1). Citrobacter braakii, as a member of the Citrobacter freundii complex, was identified in 1993 (2) and has rarely been reported as a human pathogen (3–6). The *bla*_KPC-2_ gene, as a subtype of KPC genes, has widely spread in *Enterobacteriaceae*, such as K. pneumoniae (1), Citrobacter freundii (7), C. portucalensis (8), and Escherichia coli (9) strains. However, the *bla*_KPC-2_ gene had not previously appeared in C. braakii strains. Moreover, it has been found to be carried on several plasmids to date, namely, IncR, IncP, IncFII, IncL/M, IncN, IncA, IncC, and IncX plasmids (10–12). As of 22 May 2019, 54 plasmids containing both the IncR replicon and the *bla*_KPC-2_ gene and 16 plasmids containing both the IncP6 replicon and the *bla*_KPC-2_ gene had been documented in the GenBank database, and there was no documented instance of an IncR/IncP6 hybrid plasmid (see Table S1 and S2 in the supplemental material).

10.1128/mSphere.00891-19.2 TABLE S1 Prevalence statistics of plasmids containing both the IncR replicon and the *bla*_KPC-2_ gene. Data represent statistics of plasmids containing both the IncR replicon and the *bla*_KPC-2_ gene documented as of 22 May 2019. Download Table S1, DOCX file, 0.02 MB. Copyright © 2020 Dong et al. 2020 Dong et al. This content is distributed under the terms of the Creative Commons Attribution 4.0 International license.

10.1128/mSphere.00891-19.3 TABLE S2 Prevalence statistics of plasmids containing both the IncP6 replicon and the *bla*_KPC-2_ gene. Data represent statistics of plasmids containing both the IncP6 replicon and the *bla*_KPC-2_ gene documented as of 22 May 2019. Download Table S2, DOCX file, 0.02 MB. Copyright © 2020 Dong et al. 2020 Dong et al. This content is distributed under the terms of the Creative Commons Attribution 4.0 International license.

The IncR replicon was first described in 2009 (13); since then, IncR plasmids have been increasingly reported in *Enterobacteriaceae* isolates (14). IncR replicons have also been found either as single replicons or as parts of multireplicon plasmids, which includes associations with IncA/C, IncF, IncFIIk, or nontypeable backbones (15). On the basis of prevalence statistics of plasmids containing both the IncR replicon and the *bla*_KPC-2_ gene (Table S1), we found that these plasmids usually contain multiple replicons. The *bla*_KPC-2_-carrying plasmid unnamed3 (GenBank accession no. CP027150) contains one IncR replicon from the K. pneumoniae AR_0363 strain, which was that initially reported.

IncP6 plasmids have a broad host range (16), and to date the *bla*_KPC-2_-carrying IncP6 plasmids have been found in Pseudomonas aeruginosa (16), K. oxytoca (GenBank accession no. KY913901), Enterobacter cloacae (GenBank accession no. CP018968), and C. freundii (17). Both *bla*_KPC-2_-carrying IncP6 plasmid pCOL-1 (GenBank accession no. KC609323) (18) and p10265-KPC (GenBank accession no. KU578314) (16) were recovered from P. aeruginosa strains.

In this work, we have reported the first isolation of a *bla*_KPC-2_-positive C. braakii strain. In addition, we determined the whole genomic sequence of a *bla*_KPC-2_-carrying plasmid that we have named pCRE3-KPC, which was isolated from a clinical multidrug-resistant C. braakii CRE3 strain. We compared this plasmid with the following three related plasmids: plasmid unnamed3 (GenBank accession no. CP027150), p10265-KPC (GenBank accession no. KU578314), and pCOL-1 (GenBank accession no. KC609323). Interestingly, we found that plasmid pCRE3-KPC contains both an IncR replicon and an IncP6 replicon belonging to a novel IncR/IncP6 hybrid plasmid. To the best of our knowledge, this is the first report of an IncR/IncP6 hybrid plasmid. Our results may offer insight into the horizontal transfer of resistance genes and provide an overview of plasmid diversity and evolution.

## RESULTS AND DISCUSSION

### Characterization of C. braakii CRE3.

PCR screening revealed that the multiple antimicrobial resistance genes present in C. braakii CRE3 include *bla*_KPC-2_, *bla*_TEM-1B_, *bla*_OXA-1_, *bla*_CMY-83_, *qnrB10*, and *aacC2*. Plasmid pCRE3-KPC failed to transfer to E. coli EC600 through conjugation experiments but was successfully transferred to E. coli DH5α by electroporation to generate the *bla*_KPC-2_-positive electroporant CRE3-KPC-DH5α. This result illustrates that pCRE3-KPC is a nonconjugative but mobilizable plasmid. The antimicrobial susceptibility tests showed that both the C. braakii CRE3 and E. coli electroporant CRE3-KPC-DH5α strains were highly resistant to ampicillin, piperacillin, cefuroxime, ceftriaxone, aztreonam, imipenem, meropenem, and gentamicin (Table 1). Moreover, carbapenemase was produced in both of the strains mentioned above, as revealed by the modified carbapenem inactivation method (mCIM) (19).

**TABLE 1:** Antimicrobial susceptibility profiles

Antibiotic	MIC (mg/liter)/antimicrobial susceptibility^*a*^		
	*C. braakii* CRE3	Electroporant CRE3-KPC-DH5α	E. coli DH5α
Ampicillin	≥32/R	≥32/R	≤2/R
Piperacillin	≥128/R	≥128/R	≤4/S
Cefuroxime	≥64/R	≥64/R	4/S
Ceftriaxone	≥64/R	≥64/R	≤1/S
Ceftazidime	≥64/R	4/S	≤1/S
Cefepime	≥64/R	≤1/S	≤1/S
Aztreonam	≥64/R	≥64/R	≤1/S
Imipenem	≥16/R	≥16/R	≤1/S
Meropenem	≥16/R	≥16/R	≤0.25/S
Amikacin	32/I	≤2/S	≤2/S
Gentamicin	≥16/R	≥16/R	≤1/S
Tobramycin	≥16/R	2/S	≤2/S
Ciprofloxacin	≥4/R	≤0.25/S	≤0.25/S
Levofloxacin	4/I	≤0.25/S	≤0.25/S
Nitrofurantoin	128/R	≤16/S	≤16/S

a The interpretation is derived from the Clinical and Laboratory Standards Institute guidelines (CLSI, 2018) (S, sensitive; R, resistant; I, intermediately resistant).

### Overview of plasmid pCRE3-KPC.

**FIG 1:**
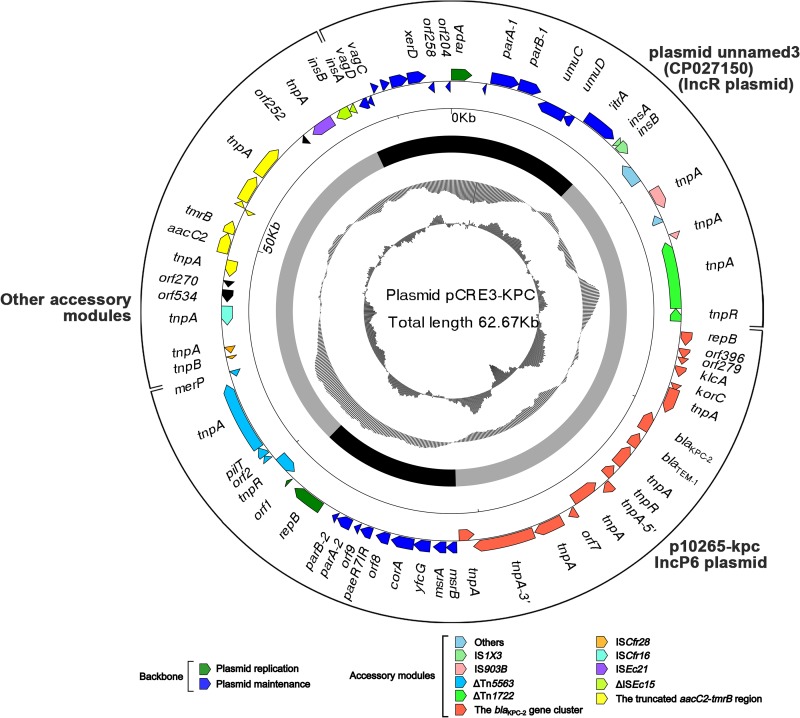
Schematic maps of plasmid pCRE3-KPC. Genes are denoted by arrows, and the backbone and accessory module regions are highlighted in black and in color, respectively. The innermost circle presents GC-skew [(G − C)/(G+C)], with a window size of 500 bp and a step size of 20 bp. The next-to-innermost circle represents GC content.

The circular DNA sequence of pCRE3-KPC is 62,673 bp in length, with mean G+C content of 56%. Furthermore, it contains 71 predicted open reading frames (ORFs) and two distinct replicons (IncR replicon *repA* and IncP6 replicon *repB*) (Table 2) (Fig. 1).

**TABLE 2:** Major features of plasmids in this work

Plasmid	Accession no. or source	Species	Inc group	Country of origin	Total length (bp)	Total no. of ORFs	Mean G+C content (%)	Accessory module(s) (resistance genes harbored)
unnamed3	CP027150	K. pneumoniae	IncR	United States	65,684	72	55	MDR region, Tn*4401a*, *aac(6′)-Ib-cr*-related region
pCRE3-KPC	This study	*C. braakii*	IncR-P6	China	62,673	71	56	*bla*_KPC-2_ gene cluster, *aacC2*-*tmrB*-related region
p10265-KPC	KU578314	P. aeruginosa	IncP6	China	38,939	46	58	*bla*_KPC-2_ gene cluster
pCOL-1	KC609323	P. aeruginosa	IncP6	Colombia	31,529	34	60	Tn*4401b*

**FIG 2:**
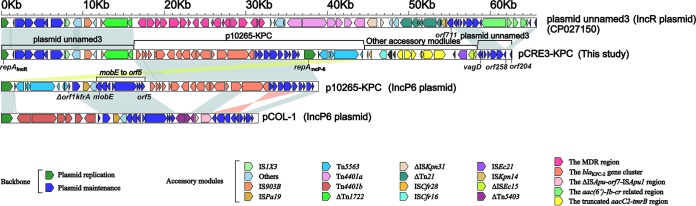
Linear comparison of pCRE3-KPC with related plasmids. A linear comparison was carried out for the complete DNA sequences of plasmids unnamed3 (GenBank accession no. KC609323), pCRE3-KPC (this study), p10265-KPC (GenBank accession no. KU578314), and pCOL-1 (GenBank accession no. KC609323). Genes are denoted by arrows. Genes, mobile elements, and other features are colored based on functional classification. Shading indicates regions of homology (>95% nucleotide identity). MDR, multidrug resistant.

Linear comparisons of plasmid pCRE3-KPC with three related reference plasmids, namely, *bla*_KPC-2_-carrying IncR plasmid unnamed3 (GenBank accession no. CP027150), p10265-KPC (a *bla*_KPC-2_-carrying IncP6 plasmid first reported in China) (16), and pCOL-1 (a *bla*_KPC-2_-carrying IncP6 plasmid, initially identified in Colombia) (18), were conducted. The detailed comparisons revealed that the overall structure of plasmid pCRE3-KPC is highly mosaic and can be divided into the following three distinct modules (Fig. 1 and 2; see also Fig. S1 in the supplemental material): (i) a first module (∼20.5 kb) that is high homologous (>98.6% identity) to plasmid unnamed3 from the K. pneumoniae AR_0363 strain reported in the United States and extends from the resolution site (*res*) of ΔTn*1722* to gene *vagD* (virulence-associated gene); (ii) a second module (∼27.8 kb) that shares >99.9% identity with plasmid p10265-KPC (16) from P. aeruginosa strain 10265 isolated in China and extends from the *bla*_KPC-2_ gene cluster to ΔTn*5563*; (iii) a third module comprising the other accessory modules (∼13.8 kb) with two novel insertion sequences (IS*Cfr28* and IS*Cfr16*), the truncated *aacC2*-*tmrB* region, IS*Ec21*, and ΔIS*Ec15.* On the basis of the study of the hybrid plasmids p675920-1 (20, 21) and pKP1034 (22), the majority of the backbone and accessory regions of unnamed3 and p10265-KPC were found to be present in pCRE3-KPC, so pCRE3-KPC may represent a combination resulting from plasmids like these. Compared to the backbone of unnamed3 and p10265-KPC, pCRE3-KPC lost part of its backbone genes (*orf711* of unnamed3, Δ*orf1* and *kfrA*, and a fragment extending from *mobE* to *orf5* of p10265-KPC) during the recombination process, suggesting that these genes may not be necessary in these plasmids. The gene functions of these plasmids are annotated in detail (see Data Set S1, S2, S3, and S4 in the supplemental material).

10.1128/mSphere.00891-19.1 FIG S1 Schematic maps of the related plasmids. The three plasmids unnamed3 (CP027150), p10265-KPC (KU578314), and pCOL-1 (KC609323) were included in the comparative analysis. Genes are denoted by arrows, and the backbone and accessory module regions are highlighted in black and in color, respectively. The innermost circle represents GC-skew [(G-C)/(G + C)], with a window size of 500 bp and a step size of 20 bp. The next-to-innermost circle represents GC content. Download FIG S1, DOCX file, 1.2 MB. Copyright © 2020 Dong et al. 2020 Dong et al. This content is distributed under the terms of the Creative Commons Attribution 4.0 International license.

10.1128/mSphere.00891-19.4 DATA SET S1 Annotations of plasmid pCRE3-KPC. Download Data Set S1, XLSX file, 0.02 MB. Copyright © 2020 Dong et al. 2020 Dong et al. This content is distributed under the terms of the Creative Commons Attribution 4.0 International license.

10.1128/mSphere.00891-19.5 DATA SET S2 Annotations of plasmid unnamed3. Download Data Set S2, XLSX file, 0.02 MB. Copyright © 2020 Dong et al. 2020 Dong et al. This content is distributed under the terms of the Creative Commons Attribution 4.0 International license.

10.1128/mSphere.00891-19.6 DATA SET S3 Annotations of plasmid p10265-KPC. Download Data Set S3, XLSX file, 0.02 MB. Copyright © 2020 Dong et al. 2020 Dong et al. This content is distributed under the terms of the Creative Commons Attribution 4.0 International license.

10.1128/mSphere.00891-19.7 DATA SET S4 Annotations of plasmid pCOL-1. Download Data Set S4, XLSX file, 0.02 MB. Copyright © 2020 Dong et al. 2020 Dong et al. This content is distributed under the terms of the Creative Commons Attribution 4.0 International license.

### Genomic comparison of the backbone regions from pCRE3-KPC and related plasmids.

The backbone of each plasmid was further divided into the replication genes and the plasmid maintenance genes, without the conjugal-transfer genes, such that the hybrid pCRE3-KPC plasmid comprised the IncR and IncP6 backbones. The resultant backbone includes two replication genes (IncR replicon *repA* and IncP6 replicon *repB*) and two sets of partitioning system *parAB* genes (Fig. 1).

The IncR backbone from pCRE3-KPC was compared with plasmid unnamed3 (an IncR plasmid; GenBank accession no. CP027150), and their backbones were found to consist of the replication genes (IncR replicon and its iterons) as well as plasmid maintenance genes (*parAB*, *umuCD*, and *vagDC*). However, two differences in their backbones were identified as follows: (i) the *orf711* gene (hypothetical protein) is deleted in pCRE3-KPC but complete in plasmid unnamed3 and (ii) the *orf258* gene (hypothetical protein) is interrupted into two parts by the insertion of the *aac(6′)*-*Ib*-*cr*-related region in plasmid unnamed3 (Fig. 1 and 2; see also Fig. S1).

Furthermore, p10265-KPC (16) and pCOL-1 (18) can be assigned to the IncP6 incompatibility group, according to replicon-based schemes. The IncP6 backbone of pCRE3-KPC was compared with those of both of the plasmids named above, and the backbones were found to comprise the replication genes (IncP6 replicon and its iterons) and plasmid maintenance genes (*kfrA*, *parABC*, the *mob* gene cluster, the *msrB*-*msrA*-*yfcG*-*corA*-*orf8* gene cluster, and *paeR7IR*). Three differences were notable among them (Fig. 1 and 2; see also Fig. S1): (i) pCRE3-KPC has lost genes (Δ*orf1* and *kfrA*) and a fragment extending from *mobE* (auxiliary protein) to *orf5* (hypothetical protein); (ii) the numbers of copies of the 17-bp tandem repeat (GCGCCTGCCTTTGAGTA) within the iterons were 11 in pCRE3-KPC, 6 in p10265-KPC, and 12 in pCOL-1; and (iii) the Δ*orf8*-*corA*-*yfcG*-*msrA*-*msrB* gene cluster was found to be inverted in pCOL-1.

### Genomic comparison of the *bla*_KPC-2_ gene region from pCRE3-KPC with those from related plasmids.

**FIG 3:**
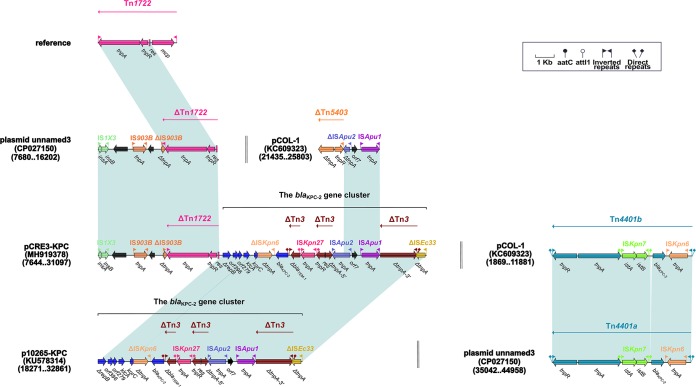
The *bla*_KPC-2_ gene region from pCRE3-KPC and comparison with the related plasmids. Genes are denoted by arrows. Mobile elements, genes, and other features are colored based on functional classification. Numbers in parentheses denote GenBank numbers and the nucleotide positions within the corresponding plasmids. Shaded regions show shared DNA regions of homology (>95% nucleotide identity). For reference, the accession number of Tn*1722* is X61367.

The *bla*_KPC-2_ gene is associated with the core *bla*_KPC_ platform (Tn*3*-IS*Kpn27*-*bla*_KPC_-ΔIS*Kpn6*) in most Chinese *Enterobacteriaceae* strains (23–25). This core platform is integrated into a ΔIS*Ec33*-associated *bla*_KPC-2_ cluster, which was initially discovered in the p10265-KPC plasmid from a P. aeruginosa strain (16). In the *bla*_KPC-2_ gene cluster of p10265-KPC, the primary genetic structure, Tn*3*-IS*Kpn27*-*bla*_KPC-2_-ΔIS*Kpn6*-*korC*-*orf6*-*klcA*-Δ*repB*, may have undergone two evolutionary events (16): (i) insertion of a Δ*bla*_TEM-1_ gene between IS*Kpn27* and the Tn*3* IRR (right inverted repeat) and (ii) disruption of the *tnpA* gene (transposase) from Tn*3*, resulting in its becoming two separate parts, an event caused by insertion of a composite transposon, IS*Apu1*-*orf7*-IS*Apu2*. The *bla*_KPC-2_-carrying pCRE3-KPC plasmid was detected in an inpatient at a tertiary care hospital in China, and the BLASTN analysis of it showed that the surrounding genetic environment of the *bla*_KPC-2_ gene in pCRE3-KPC is highly similar to that in p10265-KPC. The ΔIS*Apu1*-*orf7*-IS*Apu2* composite transposon is also present in pCOL-1, but it has not been inserted into Tn*3* and occurs downstream of ΔTn*5403*. Furthermore, the *bla*_KPC-2_ gene cluster is located downstream of ΔTn*1722*. Tn*1722*, a Tn*3*-family transposon, consists of an IRL (left inverted repeat), *tnpA*, *tnpR* (resolvase), *res*, *mcp* (methyl-accepting chemotaxis protein), and an IRR (26). ΔTn*1722* contains an IRR, *tnpA*, *tnpR*, and *res* in pCRE3-KPC, which is also present in plasmid unnamed3 (GenBank accession no. CP027150) (Fig. 3).

However, the Tn*3*-family Tn*4401* transposon has contributed to the rapid dissemination of the *bla*_KPC-2_ gene in Europe and the Americas. A number of previously reported isoforms of Tn*4401*, which differ by a 100-to-200-bp sequence upstream of *bla*_KPC-2_, are currently known (27–29). For example, Tn*4401b*, which is a Tn*4401* isoform, contains IRL, *tnpR*, *tnpA*, IS*Kpn7*, *bla*_KPC-2_, IS*Kpn6*, and IRR. Plasmid pCOL-1 (18) and plasmid unnamed3 (GenBank accession no. CP027150) originated from Colombia and the United States, respectively. The *bla*_KPC-2_ genes carried by plasmid pCOL-1 and plasmid unnamed3 are embedded in Tn*4401b* and Tn*4401a*, respectively. Compared with the complete Tn*4401b*, Tn*4401a* in plasmid unnamed3 (GenBank accession no. CP027150) has lost a 135-bp sequence upstream of *bla*_KPC-2_ (Fig. 3).

### Genomic comparison of the *aacC2-tmrB*-related region from pCRE3-KPC with those from related plasmids.

The *aacC2*-*tmrB*-related region from pCRE3-KPC is composed of ΔTn*5563*, two novel insertion sequences (IS*Cfr28* and IS*Cfr16*), the truncated *aacC2*-*tmrB* region, IS*Ec21*, and ΔIS*Ec15*. The Tn*5563* element is organized sequentially with an IRL, *tnpR*, *orf2* (hypothetical protein), *pilT* (PilT domain-containing protein), *tnpA*, *merP* (mercuric transport protein periplasmic component), *merT* (mercuric transport protein), *merR* (mercuric resistance operon regulatory protein), and an IRR. In p10265-KPC (16), Tn*5563*, which is located upstream of two consecutive backbone genes (Δ*orf1* and *kfrA*), differs from the prototype Tn*5563* from pRA2 (30) with a 286-bp insertion occurring between *merP* (mercuric transport protein periplasmic component) and *merT* (mercuric transport protein). However, ΔTn*5563* has undergone the deletion of a fragment extending from *merT* to the IRR in pCRE3-KPC (Fig. 2 and 3).

**FIG 4:**
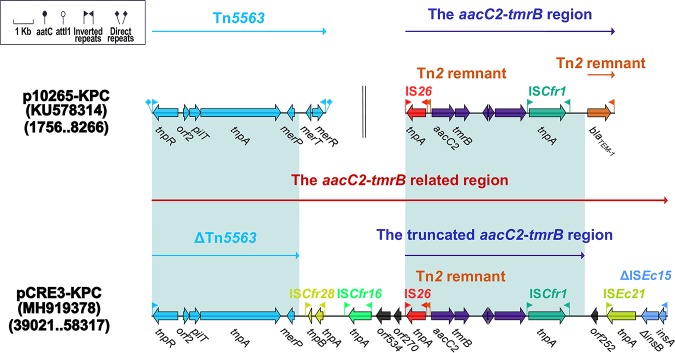
The *aacC2-tmrB*-related region from pCRE3-KPC and comparison with related plasmids. Genes are denoted by arrows. Genes, mobile elements, and other features are colored based on functional classification. Numbers in parentheses denote GenBank numbers and the nucleotide positions within the corresponding plasmids. Shaded regions indicate shared DNA regions of homology (>95% nucleotide identity). For reference, the accession number of the *aacC2*-*tmrB* region is JX101693.

In addition, two novel insertion sequences (IS*Cfr28* and IS*Cfr16*) are inserted downstream of ΔTn*5563*. IS*Cfr28*, containing two transposase genes, *tnpA* and *tnpB*, and a Tn*3* family element, is bordered by 13-bp IRs (IRL, GTCAGCCAAGAAG; IRR, CTTCTTGGCTGAC) (Fig. 4). The 1,025-bp IS*Cfr16* insertion sequence, a Tn*3* family element, is made up of a transposase gene (*tnpA*) and 13-bp IRs (IRL, TAAGCTGCGAGCG; IRR, CGCTCGCAGCTAA). The *aacC2* (aminoglycoside resistance)-*tmrB* (tunicamycin resistance) region is derived from transposon Tn*2*, and Tn*2* has undergone the following molecular evolutionary changes (31, 32): (i) the *tnpR*-*res*-*tnpA* segment of Tn*2* has been replaced by the *aacC2*-*tmrB*-*orf192*-*orf228*-*orf1182*-IS*Cfr1* module and (ii) the IS*26* insertion sequence has been inserted at the right-hand end of Tn*2*. The complete *aacC2*-*tmrB* region was discovered in pEl1573 from E. cloacae (33), and its truncated forms have been integrated into transposon Tn*6411* from the chromosome of P. aeruginosa 12939 (34). Because IS*Ec21* had inserted upstream of IS*Ec15*, this may have led to the truncation of IS*Ec15* (Fig. 4).

## MATERIALS AND METHODS

### Bacterial isolates and identification.

The clinical C. braakii CRE3 strain was isolated from a drainage sample from a patient at a tertiary care hospital in China on 5 May 2018. Bacterial identification was carried out using a Vitek compact-2 automated system (bioMérieux, France) and was confirmed by 16S rRNA sequencing (35). The genes encoding extended-spectrum β-lactamase (36), carbapenemase (37), fluoroquinolone (38), and aminoglycoside (39) were detected by PCR. All the PCR amplicons were sequenced on an ABI 3730 platform (Applied Biosystems, USA).

### Plasmid conjugal transfer.

The pCRE3-KPC plasmid was recovered from a clinical multidrug-resistant C. braakii CRE3 isolate. Conjugation experiments were carried out with cells of rifampin-resistant Escherichia coli strain EC600 as the recipient cells, and the transformation experiments were conducted using cells of E. coli DH5α Electro-Cells (TaKaRa, China) as the recipient cells for the plasmid electroporation. Plasmid pCRE3-KPC was extracted from the cells using a Qiagen Plasmid Midi kit (Qiagen, Germany). The plasmid conjugal transfer and electroporation tests were performed as described previously (40, 41).

### Antimicrobial susceptibility and carbapenemase activity detection.

Antimicrobial susceptibility testing was conducted using a Vitek compact-2 automated system (bioMérieux, France). The results were interpreted according to the CLSI (Clinical and Laboratory Standards Institute) 2018 performance standards (42). Carbapenemase activities were detected using mCIM (19).

### Sequencing and sequence assembly.

The bacterial genomic DNA extracted from the CRE3 isolate using a Wizard Genomic DNA purification kit (Promega, USA) was sequenced on the MiSeq (Illumina, USA) and the MinION (Oxford Nanopore, United Kingdom) platforms. The DNA library was constructed in accordance with a NEB Next Ultra II DNA Library Prep kit for Illumina, and the Illumina sequencing read length used was 300. The library preparations for the MinION platform were performed by the use of a rapid barcoding sequencing kit (SQK-RBK004) according to the protocol of the manufacturer (Oxford Nanopore Technologies), and the results were then loaded into the flow cell (FLO-MIN106D, Oxford Nanopore Technologies) for sequencing. Short Illumina reads were trimmed to remove poor-quality reads using Trimmomatic, and the contigs were assembled using Newbler3.0 (43). The long reads from MinION were combined with the short Illumina reads, which were subjected to hybrid assembly using SPAdesv3.11.1 (44). The hybrid assembly produced several scaffolds, and further bioinformatics analysis verified that the scaffold of the pCRE3-KPC plasmid was successfully cyclized by our in-house script. The correctness was then demonstrated by mapping the Illumina reads to the cyclized scaffold using CLC Genomics Workbench 9.0 (CLC Bio, Denmark), with an average level of read mapping coverage of 817×. The final consensus sequence obtained from CLC Genomics Workbench 9.0 was considered to represent the complete sequence of plasmid pCRE3-KPC.

### Sequence annotation and genome comparisons.

Annotation of open reading frames (ORFs) and pseudogenes was performed using RAST2.0 (45) combined with BLASTP/BLASTN searches against the UniProtKB/Swiss-Prot (46) and RefSeq (47) databases. Resistance genes, mobile elements, and other features were predicted using ResFinder3.2 (48), INTEGRALL (49), ISfinder (50), and PlasmidFinder2.1 (51) online databases. Paired-sequence comparisons and multiple-sequence comparisons were carried out using BLASTN and MUSCLE 3.8.31 (52), respectively. Gene organization diagrams were drawn in Inkscape 0.48.1 (https://inkscape.org/en/).

### Accession number(s).

The complete sequence of pCRE3-KPC was submitted to GenBank and deposited under accession number MH919378.
