# Supplementary material for: Novel IncR/IncP6 Hybrid Plasmid pCRE3-KPC Recovered from a Clinical KPC-2-Producing Citrobacter braakii Isolate
Source: mSphere. 2020 Mar 25;5(2):e00891-19. doi: 10.1128/mSphere.00891-19 (PMC7096625; doi:10.1128/mSphere.00891-19)
Supplement: TABLE S1 [file mSphere.00891-19-st001.docx]

**Table S1|** **Prevalence data statistics of plasmids containing both the IncR replicon and the *bla*_KPC-2_ gene.**

| **Plasmid** | **Accession number** | **Total length(bp)** | **Submitted_date** | **released_date** | **Source** | **Country** | **Strain** | **Replication** |
| --- | --- | --- | --- | --- | --- | --- | --- | --- |
| **pCfr-36808cz** | MG557997 | 46826 bp | 20-Nov-17 | 13-Dec-17 | hospital | Czech | Citrobacter freundii | IncR, repA(delta-repA I) |
| **pCfr-33038cz** | MG557996 | 46826 bp | 20-Nov-17 | 13-Dec-17 | hospital | Czech | Citrobacter freundii | IncR, repA(delta-repA I) |
| **pCfr-31260cz** | MG557995 | 46826 bp | 20-Nov-17 | 13-Dec-17 | hospital | Czech | Citrobacter freundii | IncR, repA(delta-repA I) |
| **pCfr-27569cz** | MG557994 | 46826 bp | 20-Nov-17 | 13-Dec-17 | hospital | Czech | Citrobacter freundii | IncR, repA(delta-repA I) |
| **pCrf-36049cz** | MF497781 | 81348 bp | 17-Jul-17 | 20-Nov-17 | hospital | Czech | Citrobacter freundii | IncR, repA(delta-repA I) |
| **pCfr-31816cz** | MF497780 | 46826 bp | 17-Jul-17 | 20-Nov-17 | hospital | Czech | Citrobacter freundii | IncR, repA(delta-repA I) |
| **pAUSMDU8141-3** | CP022698 | 107249 bp | 5-Aug-17 | 14-Aug-17 |  | Australia: Victoria | Citrobacter freundii | IncR, IncFII(K) |
| **p1761_01** | CP039975 | 282961 bp | 1-May-19 | 8-May-19 | bronchoalveolar lavage | USA:Pittsburgh | Klebsiella pneumoniae | IncR, IncFII(Yp), IncFII(K), IncFIB(K) |
| **plasmid unnamed2** | CP020500 | 73467 bp | 27-Mar-17 | 3-Apr-18 | urine | USA: Boston | Klebsiella pneumoniae | IncR, |
| **pKPC2_020037** | CP036372 | 172770 bp | 14-Jun-17 | 5-Mar-19 |  | China: Sichuan, Chengdu | Klebsiella pneumoniae | IncR, IncFII(pHN7A8) |
| **pKPC2_095080** | CP036362 | 120195 bp | 17-Feb-19 | 1-Mar-19 |  | China: Sichuan, Chengdu | Klebsiella pneumoniae | IncR, IncFII(pHN7A8) |
| **pKPC2_015093** | CP036301 | 154724 bp | 18-Feb-19 | 28-Feb-19 |  | China: Sichuan, Chengdu | Klebsiella pneumoniae subsp. Pneumoniae | IncR, IncFII(pHN7A8) |
| **pLSH-KPN148-1** | MK396843 | 227415 bp | 5-Jan-19 | 20-Feb-19 |  | China | Klebsiella pneumoniae | IncR, IncFIB(K) |
| **pKSH203-KPC** | CP034324 | 159467 bp | 5-Dec-18 | 12-Dec-18 | blood | China | Klebsiella pneumoniae | IncR, IncFII(pHN7A8) |
| **p2b2** | CP034125 | 109179 bp | 24-Nov-18 | 3-Dec-18 | respiratory tract | China: zhejiang | Klebsiella pneumoniae | IncR, IncFII(pHN7A8) |
| **unnamed3** | CP033628 | 110020 bp | 8-Nov-18 | 19-Nov-18 | rectal swab | Italy: Aosta | Klebsiella pneumoniae | IncR, IncFII(K), IncFIA(HI1) |
| **pKPC2_115069** | CP033404 | 154986 bp | 29-Oct-18 | 5-Nov-18 |  | China: Chengdu, Sichuan | Klebsiella pneumoniae | IncR, IncFII(pHN7A8) |
| **p504051-KPC** | MH477636 | 163588 bp | 11-Jun-18 | 9-Oct-18 |  | China: Zhejiang | Klebsiella pneumoniae | IncR, IncN |
| **pKPC2_L111** | CP030134 | 60307 bp | 21-Jun-18 | 28-Aug-18 | urine | Taiwan | Klebsiella pneumoniae | IncR, replication protein |
| **pKPC2_020003** | CP031720 | 154957 bp | 20-Aug-18 | 27-Aug-18 |  | China: Sichuan | Klebsiella pneumoniae | IncR, IncFII(pHN7A8) |
| **pSH9-KPC** | MH255827 | 113941 bp | 25-Apr-18 | 22-Aug-18 |  | China | Klebsiella pneumoniae subsp. Pneumoniae | IncR, |
| **pF138_3** | CP026152 | 83260 bp | 19-Jan-18 | 30-Jan-19 | blood | China: Fujian Province | Klebsiella pneumoniae | IncR, IncFII(pHN7A8) |
| **pF127_1** | CP026141 | 164501 bp | 19-Jan-18 | 30-Jan-19 | ascites | China: Fujian Province | Klebsiella pneumoniae | IncR, IncFII(pHN7A8) |
| **pF77_1** | CP026137 | 104887 bp | 19-Jan-18 | 30-Jan-19 | bile | China: Fujian Province | Klebsiella pneumoniae | IncR, IncFII(pHN7A8) |
| **pF5_1** | CP026133 | 105124 bp | 19-Jan-18 | 30-Jan-19 | sputum | China: Fujian Province | Klebsiella pneumoniae | IncR, IncFII(pHN7A8) |
| **pF1_1** | CP026131 | 164510 bp | 19-Jan-18 | 8-Aug-18 | urine | China: Fujian Province | Klebsiella pneumoniae | IncR, IncFII(pHN7A8) |
| **pKPC2_020002** | CP028541 | 177516 bp | 8-May-17 | 12-Jun-18 |  | China: Sichuan | Klebsiella pneumoniae | IncR, IncFII(pHN7A8) |
| **unnamed3** | CP023725 | 103454 bp | 26-Sep-17 | 8-Jun-18 | intraabdominal abscess | Taiwan: Taipei | Klebsiella pneumoniae | IncR, IncN, IncFII(pHN7A8) |
| **pH17-2** | CP021195 | 107793 bp | 8-May-17 | 6-Jun-18 | hospital | China: Hefei | Escherichia coli | IncR, RepA |
| **pKPC-QL24** | MH263653 | 126126 bp | 26-Apr-18 | 28-May-18 | neonate Unit | China | Klebsiella pneumoniae | IncR, IncFII(pHN7A8) |
| **pKPC2_020079** | CP029381 | 146790 bp | 15-May-18 | 21-May-18 |  | China: Sichuan | Klebsiella pneumoniae subsp. Pneumoniae | IncR, IncFII(pHN7A8) |
| **pKPC-L388** | CP029225 | 145851 bp | 10-May-18 | 30-Apr-18 | feces | China:Zhejiang | Klebsiella pneumoniae | IncR, IncFII(pHN7A8) |
| **p1642-1** | MF156695 | 165400 bp | 24-May-17 | 5-May-18 |  | China | Klebsiella pneumoniae | IncR, RepA2, RepA1 |
| **p721005-KPC** | MG764550 | 164198 bp | 5-May-18 | 8-Jan-18 |  | China | Klebsiella pneumoniae | IncR, IncN |
| **pKPC2_040035** | CP028796 | 112467 bp | 28-Feb-18 | 18-Apr-18 |  | China: Chengdu, Sichuan | Klebsiella pneumoniae | IncR, IncFII(pHN7A8) |
| **pKPC2_085072** | CP028805 | 131028 bp | 4-Apr-17 | 18-Apr-18 |  | China: Sichuan, Chengdu | Klebsiella pneumoniae | IncR, IncFII(K) |
| **pKPC2_020036** | CP028582 | 149258 bp | 14-Jun-17 | 17-Apr-18 |  | China: Sichuan, Chengdu | Klebsiella pneumoniae | IncR, IncFII(pHN7A8) |
| **unnamed3** | **CP027150** | **65684 bp** | **26-Feb-18** | **31-May-18** |  |  | **Klebsiella pneumoniae** | **IncR,** |
| **pKPC2_095084** | CP027067 | 120074 bp | 22-Feb-18 | 19-Mar-18 |  | China: Sichuan, Chengdu | Klebsiella pneumoniae | IncR, IncFII(pHN7A8) |
| **pKPC2_095649** | CP026584 | 156099 bp | 17-Nov-16 | 28-Feb-19 | Secreta | China: Sichuan | Klebsiella pneumoniae | IncR, IncFII(pHN7A8) |
| **pKPGD4** | CP025952 | 170821 bp | 12-Jan-18 | 21-Jan-18 |  | China | Klebsiella pneumoniae subsp. Pneumoniae | IncR, IncFII(pHN7A8) |
| **p187-2** | CP025468 | 129684 bp | 16-Dec-17 | 28-Dec-17 |  | China:Shanghai | Klebsiella pneumoniae | IncR, IncFII(K) |
| **p44-2** | CP025463 | 161580 bp | 16-Dec-17 | 28-Dec-17 |  | China:Shanghai | Klebsiella pneumoniae | IncR, IncFII(pHN7A8) |
| **p69-2** | CP025458 | 128563 bp | 16-Dec-17 | 28-Dec-17 |  | China:Shanghai | Klebsiella pneumoniae | IncR, IncFII(pHN7A8) |
| **pEco-36682cz** | MG557999 | 81348 bp | 20-Nov-17 | 13-Dec-17 | hospital | Czech | Escherichia coli | IncR, repA(delta-repA I) |
| **pKpn-35786cz** | MG557998 | 46826 bp | 20-Nov-17 | 13-Dec-17 | hospital | Czech | Klebsiella pneumoniae | IncR, repA(delta-repA I) |
| **unnamed2** | CP023942 | 187926 bp | 10-Oct-17 | 5-Feb-19 | Wound | Canada: BC | Klebsiella pneumoniae | IncR, IncFII(pHN7A8) |
| **pKPC-CR-HvKP4** | MF437312 | 177585 bp | 5-Jul-17 | 11-Sep-17 |  | China | Klebsiella pneumoniae | IncR, IncFII(pHN7A8) |
| **p64917-KPC** | MF168405 | 169419 bp | 26-May-17 | 9-May-19 |  | China | Klebsiella pneumoniae | IncR, IncFII(pHN7A8) |
| **p20049-KPC** | MF168404 | 151653 bp | 26-May-17 | 9-May-19 |  | China | Klebsiella pneumoniae | IncR, IncFII(pHN7A8) |
| **p12139-KPC** | MF168403 | 169424 bp | 9-May-19 | 9-May-19 |  | China | Klebsiella pneumoniae | IncR, IncFII(pHN7A8) |
| **p1068-KPC** | MF168402 | 146103 bp | 26-May-17 | 9-May-19 |  | China | Klebsiella pneumoniae | IncR, IncFII(pHN7A8) |
| **p675920-1** | MF133495 | 163995 bp | 18-May-17 | 1-Aug-17 |  | China | Klebsiella pneumoniae | IncR, IncFII(pHN7A8) |
| **pKpQIL-D2** | KY798506 | 111742 bp | 20-Mar-17 | 15-May-17 |  | United Kingdom | Escherichia coli | IncR, IncFII(K) |

* Data statistics of plasmids containing both the IncR replicon and the *bla*_KPC-2_ gene as of May 22th, 2019
