# Supplementary material for: Novel IncR/IncP6 Hybrid Plasmid pCRE3-KPC Recovered from a Clinical KPC-2-Producing Citrobacter braakii Isolate
Source: mSphere. 2020 Mar 25;5(2):e00891-19. doi: 10.1128/mSphere.00891-19 (PMC7096625; doi:10.1128/mSphere.00891-19)
Supplement: TABLE S2 [file mSphere.00891-19-st002.docx]

**Table S2| Prevalence data statistics of plasmids containing both the IncP6 replicon and the *bla*_KPC-2_ gene.**

| **Plasmid** | **Accession number** | **Total length(bp)** | **Submitted_date** | **released_date** | **Source** | **Country** | **Strain** |
| --- | --- | --- | --- | --- | --- | --- | --- |
| **pA1705-KPC** | MH909348 | 42055 bp | 14-Sep-18 | 29-Mar-19 |  | China | Klebsiella pneumoniae |
| **pKPC2_045523** | CP032895 | 43125 bp | 10-Oct-18 | 17-Oct-18 | Homo sapiens | China: Sichuan, Chengdu | Enterobacter kobei |
| **p1713-KPC** | MH624132 | 53205 bp | 13-Jul-18 | 1-Sep-18 | river sediments | China | Aeromonas taiwanensis |
| **p198-KPC** | MH624131 | 53205 bp | 13-Jul-18 | 1-Sep-18 | river sediments | China | Aeromonas taiwanensis |
| **p186-KPC** | MH624130 | 53205 bp | 13-Jul-18 | 1-Sep-18 | river sediments | China | Aeromonas taiwanensis |
| **pGSH8M-1-2** | AP019197 | 53629 bp | 1-Nov-18 | 14-Feb-19 | wastewater treatment plant effluent | Japan:Tokyo | Aeromonas caviae |
| **pGSH8-2** | AP019194 | 39071 bp | 1-Nov-18 | 14-Feb-19 | wastewater treatment plant effluent | Japan:Tokyo | Aeromonas hydrophila |
| **pCRE12-KPC** | MK050973 | 31445 bp | 14-Oct-18 | 16-Jan-19 | Patient blood | China | Citrobacter freundii |
| **pKPC2_045096** | CP028566 | 38976 bp | 4-Apr-18 | 17-Apr-18 | Sewage | China: Sichuan, Chengdu | Aeromonas hydrophila subsp. hydrophila strain |
| **plasmid: 1** | LT992437 | 40186 bp | 22-Mar-18 | 31-Mar-18 | wastewater | Germany | Citrobacter freundii |
| **pKPC-cd17** | CP026224 | 39148 bp | 19-Jan-18 | 29-Jan-18 |  | USA | Aeromonas sp. |
| **p5-KPC** | KY913901 | 40275 bp | 7-Apr-17 | 27-Jun-17 | Patient urine | China | Klebsiella oxytoca |
| **pEC542_KPC** | CP018968 | 44320 bp | 20-Dec-16 | 3-Mar-17 |  | Viet Nam: Ho Chi Minh City | Escherichia coli |
| **p10265-KPC** | KU578314 | 38939 bp | 22-Jan-16 | 30-Mar-16 | Clinical | China | Pseudomonas aeruginosa |
| **pKPC2** | KR014106 | 44451 bp | 25-Mar-15 | 31-Dec-15 |  | China | Aeromonas hydrophila |
| **pCOL-1** | KC609323 | 31529 bp | 13-Feb-13 | 26-Jul-16 | bronchial secretion | Colombia | Pseudomonas aeruginosa |

*Data statistics of plasmids containing both the IncP6 replicon and the *bla*_KPC-2_ gene as of May 22th, 2019
