# Supplementary figures and images for: Novel IncR/IncP6 Hybrid Plasmid pCRE3-KPC Recovered from a Clinical KPC-2-Producing Citrobacter braakii Isolate
Source: mSphere. 2020 Mar 25;5(2):e00891-19. doi: 10.1128/mSphere.00891-19 (PMC7096625; doi:10.1128/mSphere.00891-19)

## Supplementary Figure

**Figure S1**


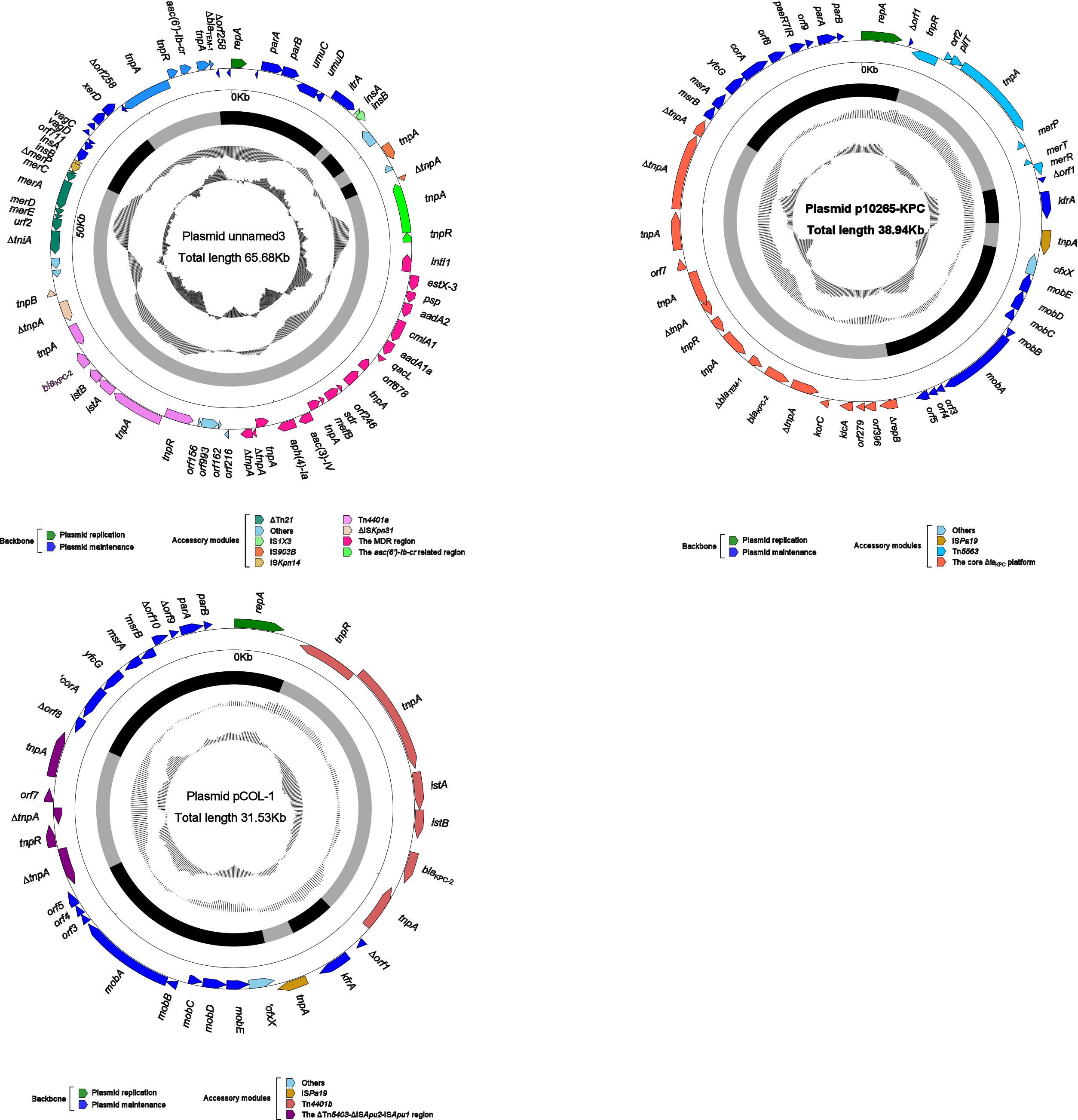

Supplement: FIG S1 [file mSphere.00891-19-sf001.docx]
